# Supplementary material for: Hepatic resistance to cold ferroptosis in a mammalian hibernator Syrian hamster depends on effective storage of diet-derived α-tocopherol
Source: Commun Biol. 2021 Jun 25;4:796. doi: 10.1038/s42003-021-02297-6 (PMC8233303; doi:10.1038/s42003-021-02297-6)
Supplement: Supplementary file 2 — Supplementary Information [file 42003_2021_2297_MOESM2_ESM.pdf]

Supplementary Information

Hepatic resistance to cold ferroptosis in a mammalian hibernator Syrian hamster depends on effective storage of diet-derived α-tocopherol

Daisuke Anegawa[1,2], Yuki Sugiura[3], Yuta Matsuoka[4], Masamitsu Sone[1], Mototada Shichiri[5], Reo Otsuka[1], Noriko Ishida[5], Ken-ichi Yamada[4], Makoto Suematsu[3], Masayuki Miura[2,], Yoshifumi Yamaguchi[1,6,7*]

Authors affiliation

[1] Hibernation Metabolism, Physiology and Development Group, Institute of Low Temperature Science, Hokkaido University, Sapporo, Hokkaido, 060-0819, Japan.

[2] Department of Genetics, Graduate School of Pharmaceutical Sciences, The University of Tokyo, Bunkyo-ku, Tokyo, 113-0033, Japan.

[3] Department of Biochemistry, Keio University School of Medicine, Shinjuku-ku, Tokyo, 160-8582, Japan.

[4] Physical Chemistry for Life Science Laboratory, Faculty of Pharmaceutical Sciences, Kyushu University, 3-1-1 Maidashi Higashi-ku, Fukuoka 812-8582, Japan.

[5] Biomedical Research Institute, National Institute of Advanced Industrial Science and Technology (AIST), 1-8-31 Midorigaoka, Ikeda, Osaka 563-8577, Japan.

[6] Global Station for Biosurfaces and Drug Discovery, Global Institution for Collaborative Research and Education (GI-CoRE), Hokkaido University, Kita-12, Nishi-6, Kita-ku, Sapporo 060-0812, Japan.

[7] Inamori Research Institute for Science Fellowship (InaRIS), 620 Suiginya-cho, Shimogyo-ku, Kyoto 600-8411, Japan.

*Corresponding author: bunbun@lowtem.hokudai.ac.jp

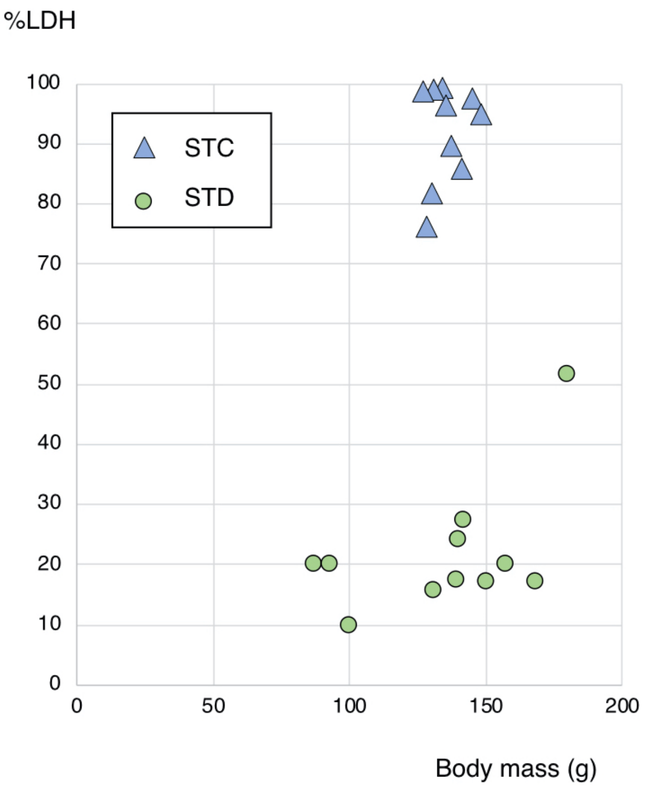

Supplementary Figure. 1

Relationship between the amount of cell death (%LDH) in hepatocytes exposed to cold culture for 48 h and body mass of the hamster used for hepatocytes isolation. Body mass did not contribute to differential susceptibility between STC and STD.

a

Lipid	Factor loading for PC1
PC(34:4)	-0.99
PC(36:6)	-0.98
PC(38:7)	-0.96
PC(34:3)	-0.94
PC(38:4)	-0.85
PC(36:4)	-0.83
PC(42:6)	-0.77
PC(36:5)	-0.72
PC(32:1)	-0.68
PC(38:6)	-0.63
PC(40:6)	-0.18
PC(38:5)	0.14
PC(34:1)	0.17
PC(34:2)	0.19
PC(36:1)	0.36
PC(36:2)	0.38
PC(40:7)	0.54

b

Lipid	Factor loading for PC1
PE(40:4)-3	-0.99
PE(38:4)	-0.99
PE(38:3)	-0.97
PE(36:4)-2	-0.96
PE(32:0)	-0.95
PE(36:5)-1	-0.93
PE(36:4)-1	-0.92
PE(36:0)	-0.90
PE(36:5)-2	-0.89
PE(34:0)	-0.88
PE(38:6)-2	-0.86
PE(34:3)-1	-0.82
PE(38:2)-3	-0.81
PE(32:1)	-0.64
PE(34:2)	-0.62
PE(36:5)-3	-0.56
PE(40:4)-2	-0.52
PE(38:2)-2	-0.43
PE(40:4)-1	-0.33
PE(34:3)-2	0.13
PE(38:5)-2	0.24
PE(38:5)-1	0.26
PE(40:6)	0.37
PE(34:1)	0.47
PE(40:5)-1	0.77
PE(40:7)	0.82
PE(40:5)-2	0.84
PE(36:3)	0.87
PE(36:1)	0.88
PE(38:6)-1	0.92
PE(38:2)-1	0.95
PE(36:2)	0.98

Supplementary Table 1. Contribution of each PLs to PC1 in PCA.

Quantified 17 PL-PC species (A) and 32 PL-PE species (B) with their factor loadings for PC1 axis in PCA.

a

	STC	STD
Water	9.2%	9.0%
Crude protein	18.8%	24.2%
Crude fat	3.9%	4.5%
Coarse fiber	6.6%	4.0%
Ash content	6.9%	6.5%
Nitrogen-free extract	54.7%	51.9%
Calorie	3291 kcal/kg	3449 kcal/kg
Vitamin A	16150 IU	15653 IU
Vitamin D3	3169 IU	2293 IU
Vitamim E	45.2 mg	208.3 mg
Vitamim K3	15.4 mg	27.4 mg
Choline	1820.0 mg	2948.0 mg
Folic acid	2.6 mg	5.6 mg
Niacin	101.7 mg	67.8 mg
Pantothenic acid	25.1 mg	41.1 mg
Biotin	0.3 mg	0.8 mg
Vitamin B1	10.9 mg	41.8 mg
Vitamin B2	8.9 mg	19.8 mg
Vitamin B6	18.9 mg	21.3 mg
Vitamin B12	0.026 mg	0.030 mg
Vitamin C	49.1 mg	1562.6 mg
Inositol	9.7 mg	741.2 mg
Carotene	1.2 mg	1.8 mg
Arginine	1.09%	1.27%
Histidine	0.46%	0.54%
Isoleucine	0.67%	0.89%
Leucine	1.36%	1.81%
Lysine	0.89%	1.09%
Methionine	0.26%	0.35%
Phenylalanine	0.84%	1.17%
Threonine	0.64%	0.80%
Tryptophan	0.21%	0.23%
Valine	0.79%	1.00%
Cysteine	0.28%	0.35%
Ca	0.98%	1.17%
Cl	0.41%	0.26%
Mg	0.26%	0.17%
P	0.80%	0.69%
K	0.97%	0.82%
Na	0.29%	0.22%
I	0.77 mg/kg	1.12 mg/kg
Fe	239.8 mg/kg	238.7 mg/kg
Co	0.08 mg/kg	0.12 mg/kg
Mn	101.2 mg/kg	68.1 mg/kg
Se	0.15 mg/kg	0.18 mg/kg
Zn	88.7 mg/kg	40.6 mg/kg
Cu	14.2 mg/kg	12.9 mg/kg

b

	STC	STD
Total FA	3.95%	4.84%
SFA	0.74%	0.94%
UFA/SFA ratio	4.33%	4.14%
MUFA/SFA ratio	1.21%	1.12%
MUFA	0.90%	1.06%
PUFA	2.31%	2.84%
HUFA	0.23%	0.35%
PUFA/MUFA ratio	2.56%	2.67%
n-3 PUFA	0.23%	0.35%
n-6 PUFA	2.08%	2.49%
n-6/n-3 ratio	9.04%	7.11%
C14:0	0.01%	0.02%
C14:1	N.D.	N.D.
C15:0	N.D.	0.01%
C16:0	0.65%	N.D.
C16:1	0.03%	0.02%
C17:0	N.D.	N.D.
C17:1	N.D.	N.D.
C18:0	0.07%	0.13%
C18:1	0.82%	0.98%
C18:2 n-6	2.08%	2.49%
C18:3 n-3	0.16%	0.26%
C20:0	0.01%	0.01%
C20:1	0.04%	0.05%
C20:5 n-3	0.03%	0.04%
C22:0	N.D.	0.01%
C22:1	0.01%	0.01%
C22:5	N.D.	N.D.
C22:6 n-3	0.04%	0.05%
C24:0	N.D.	N.D.
C24:1	N.D.	N.D.

Supplementary Table 2. Composition of diets used in this study.

Composition of ingredient according to information provided by the manufacture (A). Fatty acids composition analyzed by gas chromatography (B). SFA: saturated fatty acid. UFA: unsaturated fatty acid. MUFA: monounsaturated fatty acid. N.D. ; Not detected.
