# Supplementary material for: Hepatic resistance to cold ferroptosis in a mammalian hibernator Syrian hamster depends on effective storage of diet-derived α-tocopherol
Source: Commun Biol. 2021 Jun 25;4:796. doi: 10.1038/s42003-021-02297-6 (PMC8233303; doi:10.1038/s42003-021-02297-6)
Supplement: Supplementary file 3 — Description of Additional Supplementary Files [file 42003_2021_2297_MOESM3_ESM.pdf]

Description of Additional Supplementary Files

File name: Supplementary Data 1
Description: Raw Data for Figure 1.

File name: Supplementary Data 2
Description: Raw Data for Figure 2.

File name: Supplementary Data 3
Description: Raw Data for Figure 3.

File name: Supplementary Data 4
Description: Raw Data for Figure 4.
